# ISCEV guide to visual electrodiagnostic procedures

**DOI:** 10.1007/s10633-017-9621-y

**Published:** 2018-02-03

**Authors:** Anthony G. Robson, Josefin Nilsson, Shiying Li, Subhadra Jalali, Anne B. Fulton, Alma Patrizia Tormene, Graham E. Holder, Scott E. Brodie

**Affiliations:** 1 Department of Electrophysiology, Moorfields Eye Hospital, 162 City Road, London, UK; 2 Institute of Ophthalmology, University College London, London, UK; 3 Department of Clinical Neurophysiology, Sahlgrenska University Hospital, Göteborg, Sweden; 4 Southwest Hospital, Southwest Eye Hospital, Third Military Medical University, Chongqing Institute of Retina, Chongqing, China; 5 Srimati Kanuri Santhamma Centre for Vitreoretinal Diseases, Jasti V. Ramanamma Childrens’ Eye Care Centre, L V Prasad Eye Institute, Hyderabad, India; 6 Department of Ophthalmology, Boston Children’s Hospital, Boston, USA; 7 Department of Neurosciences, Ophthalmic Clinic, Padova University, Padova, Italy; 8 National University of Singapore, National University Hospital, Singapore City, Singapore; 9 The Mount Sinai Hospital, New York Eye and Ear Infirmary of Mount Sinai, New York, USA

**Keywords:** ISCEV standards, Clinical electrophysiology, Electrooculogram (EOG), Electroretinogram (ERG), Pattern ERG, Multifocal ERG (mfERG), Visual evoked potential (VEP), Optic neuropathy, Maculopathy, Retinopathy

## Abstract

Clinical electrophysiological testing of the visual system incorporates a range of noninvasive tests and provides an objective indication of function relating to different locations and cell types within the visual system. This document developed by the International Society for Clinical Electrophysiology of Vision provides an introduction to standard visual electrodiagnostic procedures in widespread use including the full-field electroretinogram (ERG), the pattern electroretinogram (pattern ERG or PERG), the multifocal electroretinogram (multifocal ERG or mfERG), the electrooculogram (EOG) and the cortical-derived visual evoked potential (VEP). The guideline outlines the basic principles of testing. Common clinical presentations and symptoms are described with illustrative examples and suggested investigation strategies.

## Introduction

Clinical electrophysiological testing of the visual system incorporates a range of tests based upon the recording of electrical potentials evoked by visual stimuli, using electrodes situated on the surface of the eyes, the peri-orbital skin or scalp. The tests are noninvasive and provide an objective indication of function relating to different locations and cell types within the visual system. This document developed by the International Society for Clinical Electrophysiology of Vision (ISCEV) provides an introduction to standard visual electrodiagnostic procedures in widespread use and describes the common clinical indications for which these tests are applicable. Detailed specifications for each procedure may be found in the appropriate ISCEV standards [1–5]. The basic principles of electrodiagnostic testing are outlined in this document, but the document is not intended to be prescriptive or to address every clinical scenario and is not a mandate for specific procedures on individual patients. Clinical electrophysiological testing has the greatest utility when performed in conjunction with clinical assessment by specialist eye care professionals. Clinical context is essential to enable appropriate clinical management.

This guideline describes the basic methods and underlying principles of testing for each of the standard tests including the full-field flash electroretinogram (ERG), the pattern electroretinogram (pattern ERG or PERG), the multifocal electroretinogram (mfERG), the electrooculogram (EOG) and the cortical-derived visual evoked potential (VEP). The principal focus is to place these tests in clinical context. Common clinical presentations and symptoms are described with illustrative examples and suggested investigation strategies.

## The electrophysiological tests

ISCEV publishes and regularly updates standards for clinical tests of the visual system. The most recent publications are listed on the ISCEV Web site www.iscev.org/standards and are freely accessible. In addition to these basic tests, extended protocols may support differential diagnosis or functional monitoring. Below is a brief description of normal waveforms resulting from the ISCEV standard tests and the physiologic implications of abnormal responses. Users should consult the relevant standard or extended protocol for detailed testing protocols.

### The full-field ERG

**Fig. 1:**
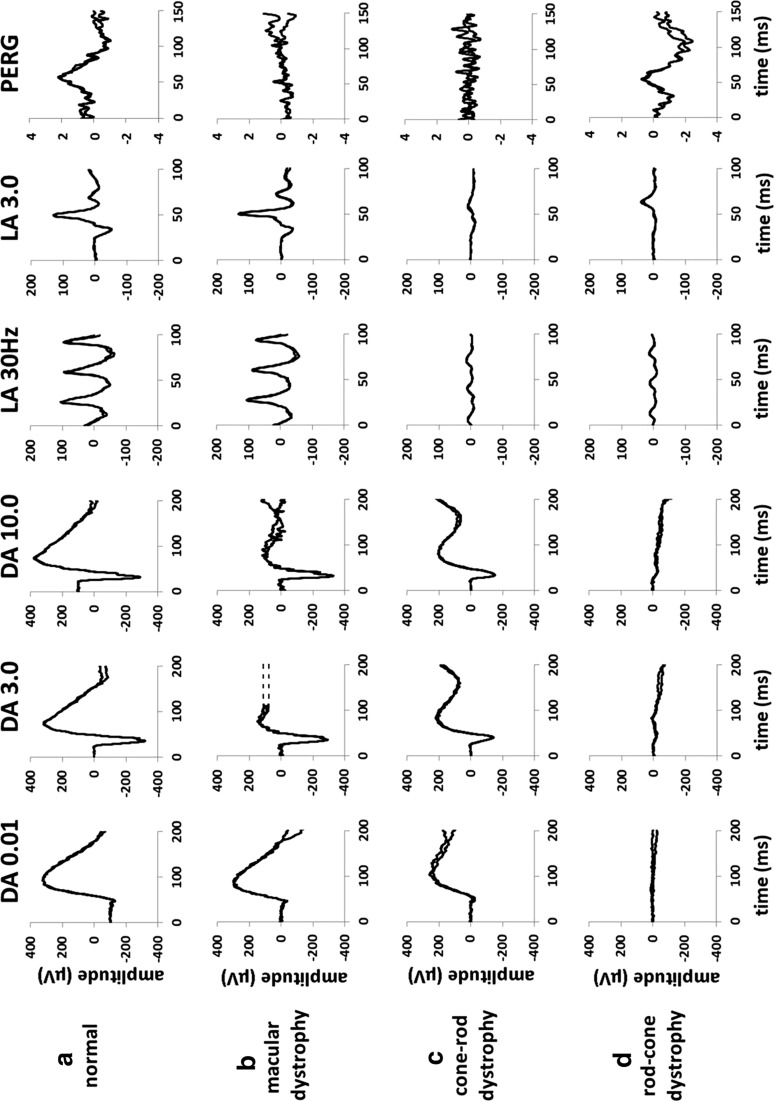

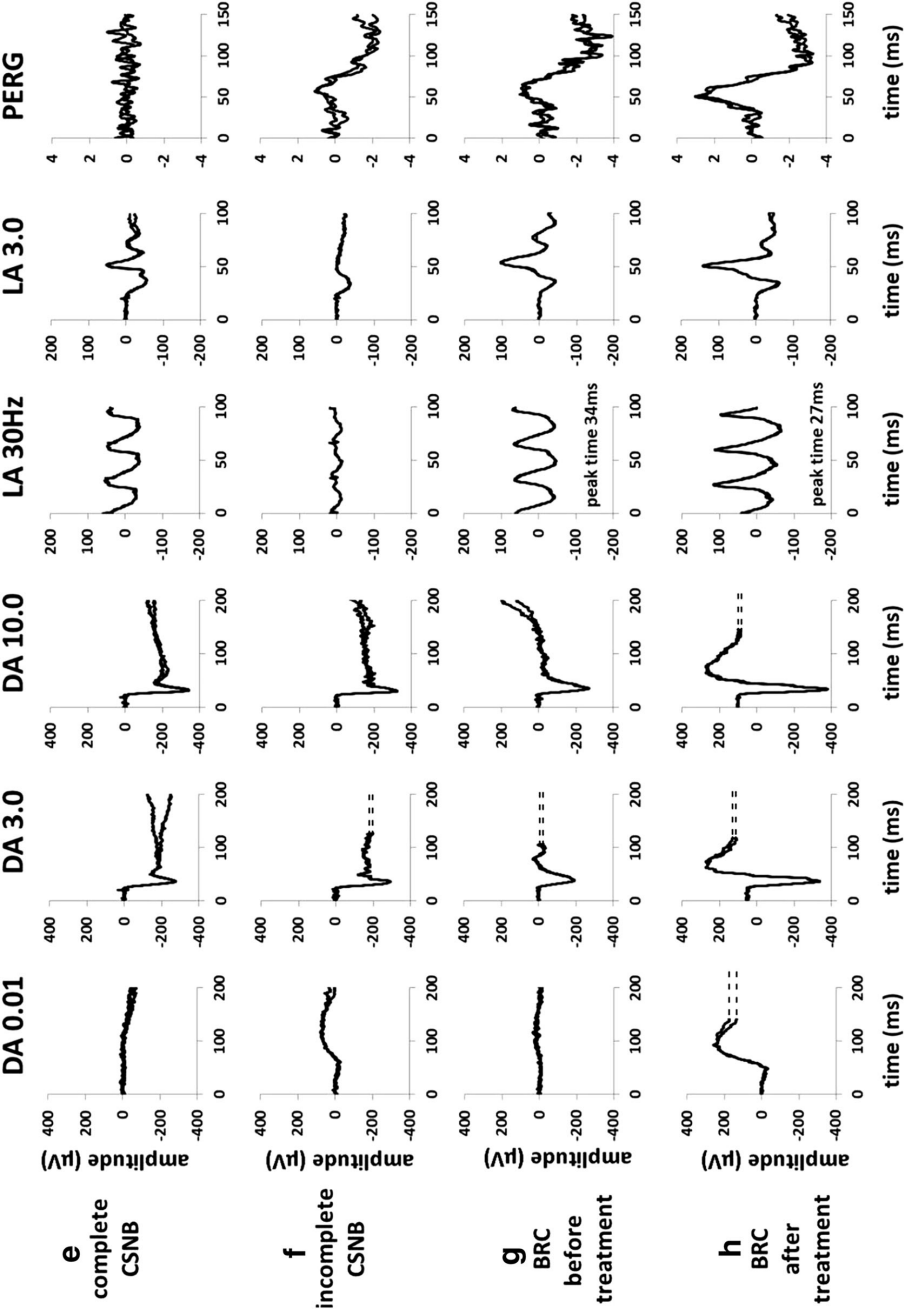
Representative full-field and pattern ERGs in a normal subject (**a**), in a case of macular dystrophy (**b**), cone-rod dystrophy (**c**), rod-cone dystrophy with relative sparing of macular function (**d**), complete CSNB (**e**), incomplete CSNB (**f**) and birdshot retinochoroidopathy (BRC) before treatment (**g**) and after treatment illustrating full recovery of the ERG and PERG (**h**). Recordings showed a high degree of inter-ocular symmetry except in BRC (data from other eye are not shown). Note there is a 20-ms pre-stimulus delay in all single flash ERG recordings. Two responses for each stimulus condition are superimposed to illustrate reproducibility. Broken lines replace blink artefacts occurring after the ERGs, for clarity

The ISCEV standard full-field ERGs (Fig. 1a) are global responses of the retina to brief flashes of light and provide an assessment of generalized retinal function under light- and dark-adapted conditions. A ganzfeld (German for “whole field”) stimulator, which provides a uniformly illuminated field, is used to deliver a range of flash stimuli that evenly illuminate the maximal area of retina. The ERGs are recorded with electrodes in contact with the cornea or conjunctiva or with skin electrodes attached to the lower eyelids. Several types of corneal electrode may be used including contact lens, fiber, jet and gold foil electrodes. The pupils are dilated to maximize retinal illumination and to minimize inter-subject and inter-visit variability. Reliable interpretation of recordings requires comparison with electrode-specific and age-matched normative data. The normal test–retest variability of ERG parameters is also an important consideration if used to monitor disease progression or the safety or efficacy of treatments.

The ISCEV standard protocol includes dark-adapted (DA) recordings after 20-min dark adaptation to flash strengths of 0.01, 3.0 and 10.0 cd s m^−2^ (DA 0.01; DA 3.0; DA 10.0). The weak flash (DA 0.01) ERG arises in the inner retinal rod bipolar cells and is the only standard test that selectively monitors rod system function. Abnormality of the DA 0.01 ERG can be caused by either rod photoreceptor dysfunction or selective dysfunction occurring post-phototransduction or at the level of the inner retinal rod bipolar cells. The DA 3.0 (standard flash) and DA 10.0 (strong flash) ERGs have input from both rod and cone systems, but the DA rod system contribution dominates in a normal retina. Approximately the first 8 ms of the cornea-negative a-wave reflects rod hyperpolarizations, and as the a-wave in the DA 10.0 ERG is of shorter peak time and larger than in the DA 3.0 ERG, it provides a better measure of rod photoreceptor function. The subsequent cornea-positive b-wave arises largely in the rod On-bipolar cells and reflects function that is post-phototransduction. Thus, the DA strong flash ERG enables localization of dysfunction to the rod photoreceptors (a-wave reduction and concomitant b-wave reduction) or to a level that is post-phototransduction or inner retinal (sparing of the a-wave with b-wave reduction). The DA oscillatory potentials (OPs) are small high-frequency components normally visible on the rising limb of the DA 3.0 and DA 10.0 ERG b-waves and are thought to reflect amacrine cell signaling. Reduction in the OPs is often associated with other ERG abnormalities but may occur selectively in some disorders. The cone system contribution to both DA ERG a- and b-waves is minor in a normal retina but can be of greater significance in patients with disease primarily or exclusively affecting the rod system.

Standard light-adapted (LA) ERGs provide two measures of generalized cone system function; both are obtained to a flash strength of 3.0 cd s m^−2^, after a standard period of 10-min light adaptation in the Ganzfeld with a constant background luminance of 30 cd m^−2^. A 30 Hz flash stimulus, superimposed on the background, is used to elicit the LA 30 Hz flicker ERG, generated largely by post-receptoral retinal structures. The single flash cone (LA 3.0) ERG consists mainly of a- and b-waves. The LA 3.0 ERG a-wave arises in the cone photoreceptors and Off-bipolar cells; the b-wave is dominated by a combination of cone On- and Off-bipolar cell activity, and a reduced b/a ratio suggests cone system dysfunction that is post-phototransduction or post-receptoral.

The full-field ERG enables the distinction between generalized outer and inner retinal dysfunction and predominant rod or cone system dysfunction. Symptoms and/or clinical signs may suggest a retinopathy, but the presence, severity and nature of retinal dysfunction cannot always be inferred from the clinical findings and ERGs can help differentiate between a wide range of disorders when appropriately placed in clinical context (see below and Table 1). It is stressed that the full-field ERG is largely generated by the retinal periphery and there is minimal contribution from the macula. Electrophysiological assessment of macular function requires the use of different techniques such as the pattern ERG or multifocal ERG.

### The pattern ERG

The ISCEV standard PERG is derived largely from the macular retinal ganglion cells and complements the full-field ERG, in differentiating between maculopathy and generalized retinopathy. PERG also enables a more meaningful evaluation of a VEP, to exclude a macular cause of VEP abnormality and to provide an additional assessment of retinal ganglion cell involvement (see below). The PERG is recorded to an alternating high-contrast checkerboard using a corneal electrode. PERGs are attenuated by poor refraction and ocular media opacity, and care must be taken to optimize the optical quality of the checkerboard stimulus; for this reason, contact lens electrodes are not suitable.

**Fig. 2:**
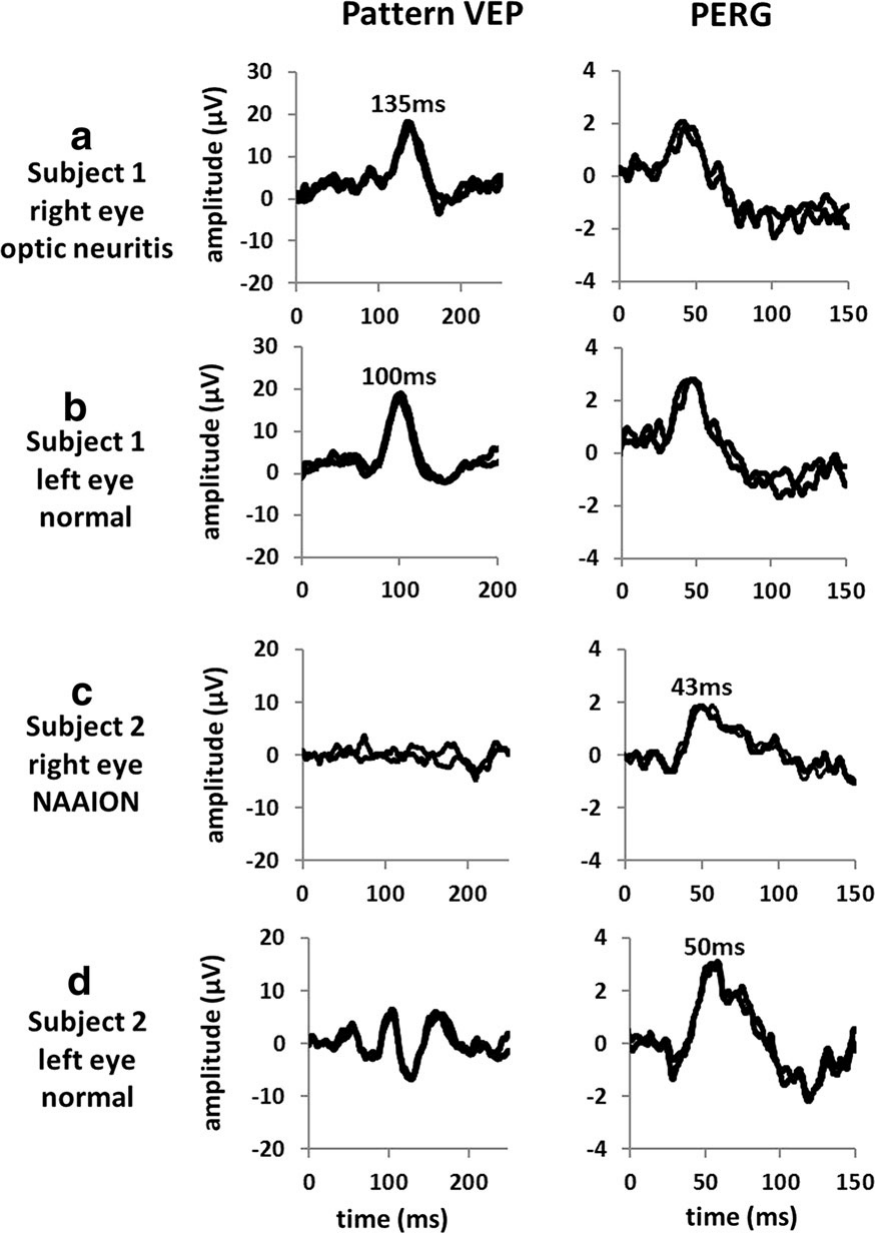
Representative pattern-reversal VEPs and PERGs in the affected (**a**, **c**) and fellow (**b**, **d**) eyes in a patient with non-acute optic neuritis (Subject 1; **a**, **b**) and in an elderly patient with a severe non-arteritic anterior ischemic optic neuropathy (Subject 2; **c**, **d**). The P100 component of the pattern VEP in optic neuritis shows a 35-ms delay compared with the normal fellow eye, without significant amplitude reduction, consistent with optic nerve conduction delay; pattern ERGs are normal in this case and reveal no evidence of macular or retinal ganglion cell dysfunction. The pattern VEP P100 component in **c** is undetectable, and PERG shows a reduced N95:P50 ratio and shortening of P50 peak time (inter-ocular difference 7 ms) compared with the fellow eye, indicating severe optic nerve dysfunction with retinal ganglion cell involvement. Two responses for each stimulus condition are superimposed to illustrate reproducibility

The transient PERG has two major components of diagnostic value: a positive polarity P50 and a negative polarity N95 (Figs. 1a and 2). Both components reflect macular retinal ganglion cell function, but there is an additional more distal retinal contribution to the P50 component. Both P50 and N95 depend on the function of the macular cones, and P50 reduction and/or delay can characterize macular dysfunction. Selective reduction in N95 with preservation of P50 suggests dysfunction at the level of the retinal ganglion cells. In severe or chronic retinal ganglion cell dysfunction, there may be P50 reduction, but in such circumstances P50 usually shortens in peak time, reflecting loss of the retinal ganglion cell contribution to P50. Preservation of P50 helps to establish the effective stimulus quality and contrast of the checkerboard in patients who may have poor visual acuity for reasons other than maculopathy. Comparison of responses to a standard and additional large-field stimulus may help characterize the area of macular dysfunction, although spatial resolution is lower than for the mfERG.

### The multifocal ERG

**Fig. 3:**
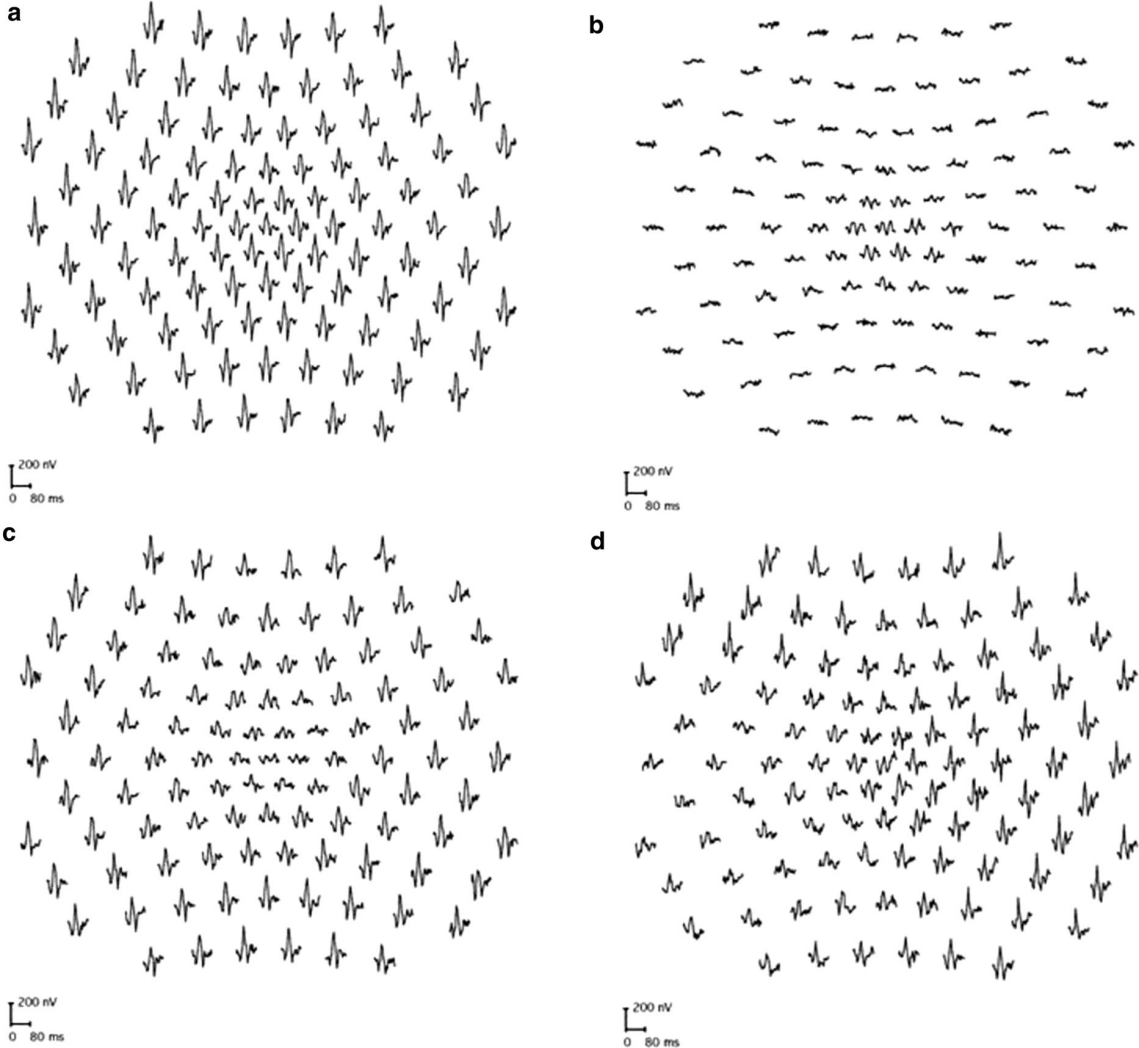
Multifocal ERGs recorded to a 103-element stimulus array in a representative normal subject (**a**), in a case of retinitis pigmentosa showing relative sparing of central macular function (**b**), in a case of macular dystrophy showing reduction over a central area (**c**) and in a patient with an eccentric nasal area of retinal dysfunction consistent with an enlarged blind spot extending inferiorly in the right eye (**d**). MfERGs in cases **a**–**c** showed a high degree of inter-ocular symmetry; abnormalities were unilateral in **d**. Traces are shown in retinal view

The ISCEV standard mfERG (Fig. 3a) provides a measure of cone system function over 61 or 103 discrete hexagonal retinal areas, within the central 40°–50° of the posterior pole centered on the macula. The hexagons of the ISCEV standard stimulus array are scaled to elicit comparable response amplitudes from each stimulus region, resulting in larger hexagons with increasing eccentricity. Reliable recording requires good patient fixation, and corneal electrodes are required as signals are small.

Each hexagonal stimulus element is modulated rapidly to display white or black frames according to an irregular but predetermined binary sequence known as a “pseudorandom” or “m-sequence.” The signal associated with a particular hexagon is extracted from a single continuous recording from each eye, using automated cross-correlation analysis. The responses can be mathematically stratified into components associated with single illumination events (the first-order kernel), used for ISCEV standard testing. The optical quality of the stimulus is important, and patients should be optimally refracted and must fixate accurately on a central target or cross-hairs throughout the recording period. There is a compromise between spatial resolution (smaller, more numerous hexagons) and the recording duration necessary to obtain responses with a satisfactory signal-to-noise ratio. There are two major response components; an early negative polarity N1 component is derived from cone bipolar cells with a cone photoreceptor contribution and a later positive polarity P1 component that arises in cone bipolar cells.

The spatial resolution of the mfERG is better than for the PERG and full-field ERGs, and this enables improved characterization of focal central, annular, hemifield or discrete paracentral areas of posterior pole dysfunction, but reliable recording requires good patient fixation. If the area of dysfunction shows reasonably good radial symmetry, interpretation may be facilitated by averaging waveforms from all the hexagons in each concentric ring in the stimulus pattern (ring-averaging). Illustrative examples of mfERG recordings are shown in a case of retinitis pigmentosa (RP) with central macular sparing (Fig. 3b), in macular dystrophy (Fig. 3c) and in a patient with an enlarged blind spot (Fig. 3d). The mfERG is also a useful adjunct to the VEP and is less affected by optical factors than the PERG; there is no retinal ganglion cell contribution to the mfERG, and a normal response excludes primary macular photoreceptor dysfunction as cause of VEP abnormality or central visual loss. However, in some conditions such as cystoid macular edema (CME), the mfERG may be preserved or less affected than the PERG.

### The electrooculogram

The ISCEV standard EOG is used to assess generalized retinal pigment epithelium (RPE) function. There is a potential difference between the apical and basal surfaces of the RPE that results in a dipole across the eye, with the cornea being positive with respect to the back of the eye. This potential difference, the standing potential of the eye, is recorded using skin electrodes placed at the medial and lateral canthus of each eye during uniform 30-degree horizontal saccades, made periodically during dark and then light adaptation. During the standard 15-min period of dark adaptation, there is a fall in the recorded standing potential, typically reaching a minimum at 10–15 min, referred to as the dark trough (DT). The dark phase is followed by a 15-min period of continuous light adaptation to a standard white background (100 cd m^−2^), provided by a Ganzfeld stimulator. Following light onset, there is an increase from the standing potential resulting in the EOG light peak (LP). The LP/DT ratio (Arden ratio) provides a measure of the generalized function of the RPE/photoreceptor complex. The development of a normal EOG light peak requires not only a normally functioning RPE, but also normally functioning rod photoreceptors, with the degree of EOG abnormality broadly corresponding to the degree of rod-derived ERG abnormality. An EOG assessment of generalized RPE/photoreceptor function is most useful when interpreted in the context of normal or only mildly subnormal rod-mediated ERG findings. In the presence of severe rod dysfunction from any cause, the EOG will be abnormal, and not additionally informative about the function of the RPE. Common causes of generalized RPE dysfunction are outlined below.

### Visual evoked potentials

The ISCEV standard VEPs provide an important objective test in the investigation of suspected optic nerve disease or post-retinal visual pathway dysfunction. The VEPs are electrical potentials recorded from the scalp derived from electrical currents generated in the visual cortex in response to visual stimulation. The VEP indicates the function of the entire visual pathway from the retina to area V1 of the visual cortex and primarily reflects the central retinal projection to the occipital poles. Recording electrodes are positioned on the scalp according to anatomical landmarks using a standardized “International 10/20 system” measurement method. The recording montage includes at least one occipital electrode (Oz) referred to a mid-frontal reference (Fz). Computerized signal averaging is used to extract the time-locked VEP from spontaneous brain activity (the electroencephalogram or EEG).

The ISCEV standard for VEP testing describes three stimulus modalities: pattern-reversal, pattern onset–offset and diffuse flash stimulation. A reversing checkerboard is used to record the pattern-reversal VEP, generally most useful for the assessment of optic nerve function, but requiring an adequate level of fixation and compliance. The normal pattern-reversal VEP has a prominent positive component at approximately 100 ms (P100; Fig. 2), although normal ranges differ and are age and laboratory dependent. Pattern onset–offset (pattern appearance) stimulation is less commonly used in the diagnosis of optic nerve disease than pattern reversal, but has the advantage of being less affected by nystagmus. Flash VEPs are generally less sensitive to dysfunction than pattern VEPs, but may be used in young children or when patients cannot fixate or comply with testing. They are also useful in the presence of media opacity when the use of stronger non-standard flashes may be helpful to establish the integrity of the visual pathway. There is wider variability in normal ranges than for pattern VEPs, and an inter-ocular comparison is often most useful. Flash VEPs may occasionally reveal abnormalities in the presence of normal pattern VEPs, and this can occur in rare cases of optic neuritis, in some cases of optic nerve sheath pathology or due to unsuspected retinopathy.

Multichannel VEPs, in excess of the current ISCEV standard, are needed to detect optic nerve misrouting or to detect and characterize chiasmal or retrochiasmal dysfunction. Multichannel flash VEPs can also reveal the visual pathway misrouting associated with albinism in children, but flash VEPs are usually normal in adults with albinism.

The timing, amplitude and waveform shape of the P100 component are used to evaluate pattern-reversal VEPs, which provide an important objective test in the investigation of suspected optic nerve disease or post-retinal visual pathway dysfunction. However, abnormalities are not specific and can reflect, for example, optic nerve or macular dysfunction and can also be caused by poor compliance or sub-optimal refraction. Reliable interpretation of pattern VEP abnormality usually requires complementary assessment to exclude a macular cause. Similarly, a flash ERG may exclude a retinal cause of flash VEP abnormality. There are numerous causes of optic nerve disease, and VEPs may suggest or support a suspected diagnosis when interpreted in clinical context. Common causes of optic neuropathy are outlined below.

## Clinical indications for visual electrophysiology

**Fig. 4:**
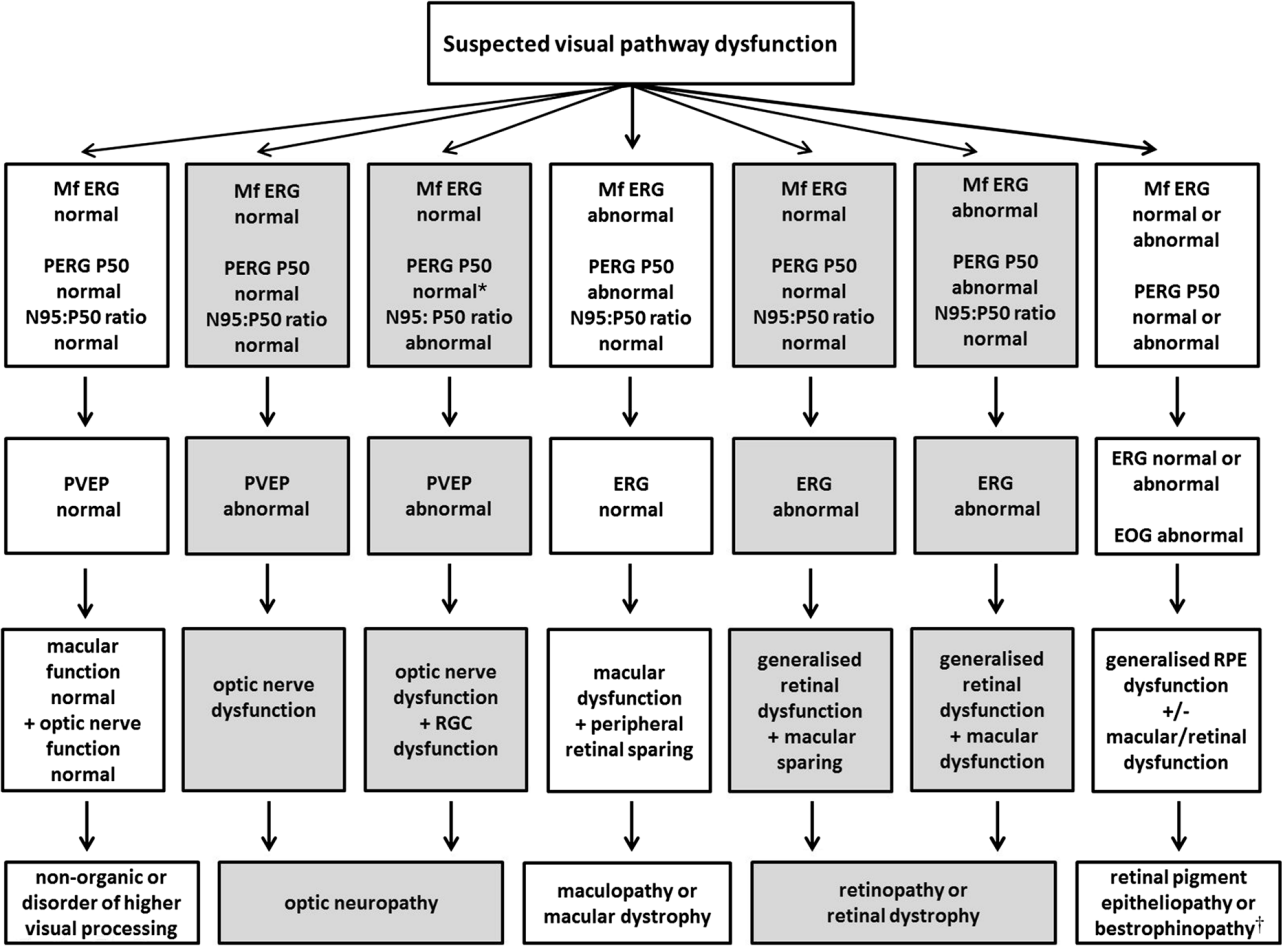
Suggested test strategy for cases of suspected visual pathway dysfunction, illustrating how complementary tests can localize dysfunction within the visual system. Asterisk (*): in cases of retinal ganglion cell dysfunction, the PERG N95:P50 ratio is subnormal, but in severe disease P50 may additionally show reduction with shortening of peak time. Dagger (†): bestrophinopathies; Best disease is associated with a normal ERG and abnormal EOG; autosomal recessive bestrophinopathy causes severe EOG reduction and later onset progressive retinopathy with relatively mild ERG abnormality; in ADVIRC, the EOG is abnormal and the ERG abnormal. See Table 1 for details. After; [6, 7]

Symptoms, signs and circumstances that frequently prompt referral for visual electrophysiology are outlined below, with selected examples illustrated in Figs. 1, 2 and 3, chosen to illustrate the underlying principles of testing. Accurate localization of dysfunction within the visual pathway may require complementary testing with different techniques, and a suggested test strategy is outlined in Fig. 4. It is stressed that multiple tests may not be needed in all patients and that electrophysiological findings and accurate diagnosis require interpretation in the context of the clinical findings. A comprehensive list of all conditions that may prompt visual electrophysiological examination is beyond the scope of this guideline, but diagnoses that commonly benefit from testing and typical findings are summarized in Table 1.

### Visual acuity loss

Visual acuity loss may be caused by inherited and acquired causes of maculopathy (with or without retinopathy), optic nerve and visual pathway disease, but this may not be obvious on clinical grounds alone and the distinction is enabled by electrophysiological testing.

#### Retinal and RPE disorders

The pattern ERG and mfERG may be used to assess the severity of macular dysfunction (Figs. 1 and 3) in the presence of fundus abnormality or used to detect dysfunction in occult cases of maculopathy or macular dystrophy. If there is visible evidence of maculopathy on fundus examination, a full-field ERG will determine whether there is peripheral retinal involvement, e.g., differentiating between macular dystrophy (normal full-field ERG; Fig. 1b) and cone and cone-rod dystrophy (see below and Fig. 1c). Common reasons for referral include bull’s eye lesions, which may be associated with macular dystrophy, cone or cone-rod dystrophy, or acquired dysfunction, for example, caused by hydroxychloroquine toxicity. In Stargardt disease (ABCA4 retinopathy), the most common monogenic cause of inherited macular/retinal dystrophy, there is usually visible maculopathy and fleck lesions (not always present in children or early disease) and ERGs establish whether dysfunction is confined to the macula or whether there is generalized cone or cone and rod involvement.

Rapid loss of visual acuity may occur in acquired disorders such as paraneoplastic (carcinoma associated retinopathy; CAR) or autoimmune retinopathy (AIR), which are often without fundus abnormality at presentation and are typically associated with pronounced rod and cone photoreceptor dysfunction, evident on ERG testing. In cases of vitelliform macular lesions, an ERG and EOG are indicated; Best vitelliform macular dystrophy is characterized by a severely reduced EOG light peak to dark trough ratio in the absence of ERG abnormality, confirming generalized RPE dysfunction and largely excluding other disorders that may resemble Best disease on fundus examination, including some pattern dystrophies such as adult-onset vitelliform macular dystrophy.

#### Optic nerve/post-retinal disorders

In the absence of obvious fundus abnormality, the pattern VEP in combination with a PERG or mfERG distinguishes optic nerve dysfunction from occult macular disease. The pattern VEP is usually abnormal in macular disease, and PERG P50/central mfERG preservation largely excludes macular dysfunction as a cause of pattern VEP abnormality.

Acute visual acuity loss with pain on eye movement is typical of optic neuritis, and VEPs are typically delayed in keeping with demyelination (Fig. 2), with or without amplitude reduction; the VEP abnormality usually persists even if visual acuity improves. VEP abnormalities may occur in an asymptomatic eye and in visually asymptomatic patients with multiple sclerosis, consistent with subclinical demyelination. Approximately 35% of patients with optic nerve demyelination manifest a reduced PERG N95:P50 ratio, in keeping with retrograde involvement of the retinal ganglion cells and occurring a minimum of 4–6 weeks after presentation, although this can occur in any form of optic neuropathy. A sudden painless and irreversible loss of vision is typical of non-arteritic anterior ischemic optic neuropathy (NAION), and unlike demyelination, pattern VEPs typically show amplitude reduction without significant delay (Fig. 2). In arteritic anterior ischemic optic neuropathy (AAION), there is usually severe visual loss and gross VEP abnormality. Leber hereditary optic neuropathy (LHON) typically presents with sudden sequential, painless visual loss, and pattern VEPs are usually undetectable or severely abnormal at presentation; PERG P50 amplitude is typically normal providing fixation is adequate, but there may be marked reduction in N95 in the acute stages, in keeping with primary ganglion cell dysfunction.

Compressive lesions of the visual pathways are associated with progressive or insidious visual acuity loss, although if unilateral this may be noticed suddenly by the patient. If a unilateral optic nerve lesion is anterior to the optic chiasm, there will be unilateral pattern VEP abnormality. Localization of dysfunction posterior to the optic nerves requires multichannel VEP recordings. Chiasmal dysfunction results in a “crossed asymmetry,” such that the VEP from each eye is abnormal over a different hemisphere. Retrochiasmal dysfunction results in an “uncrossed” asymmetry such that monocular VEPs from both eyes are abnormal over the same hemisphere. Progressive visual loss is also a feature of dominant optic atrophy and nutritional optic neuropathies such as that caused by vitamin B12 deficiency. Toxic etiology includes ethambutol, methyl-alcohol poisoning (also associated with retinopathy) and rare cases of tobacco toxicity. Visual loss may also result from injury to the occipital cortex usually resulting in both pattern and flash VEP waveform degradation or distortion.

#### Non-organic visual loss

In cases of unexplained or suspected “functional” visual loss, normal electrophysiology helps to exclude an organic cause. A well-formed pattern-reversal VEP is incompatible with a visual acuity of approximately 6/36 or worse, although care must be taken to ensure adequate patient compliance during testing. Flash VEPs are usually normal, and even if there is dysfunction with non-organic overlay, it is difficult to reconcile a detectable flash VEP with “no perception of light” vision. The significance of pattern VEP abnormality depends on the results of macular testing with PERG P50 or mfERG, and the importance of flash VEP abnormality may similarly depend on the absence of significant full-field ERG abnormality. Normal visual electrophysiology does not preclude the presence of underlying organic disease, and particular caution must be exercised if there is a possibility of higher cortical dysfunction.

### Night blindness

Night blindness (nyctalopia) can result from generalized rod system dysfunction, and this may be confirmed or excluded using a full-field ERG. The DA 3.0 and DA 10.0 ERGs enable localization of dysfunction to the rod photoreceptors (a-wave reduction and concomitant b-wave reduction) or to a level that is post-phototransduction or inner retinal (sparing of the a-wave; b-wave reduction).

#### Night blindness due to rod photoreceptor dysfunction

In progressive retinal degenerations such as retinitis pigmentosa, which in mild cases may be associated with a normal or near-normal fundus appearance, there is ERG evidence of a rod-cone dystrophy (Fig. 1d). The severity of generalized retinal dysfunction in RP varies, but there may be preserved visual acuity and relative preservation of macular function until the late stages in many cases, as revealed by PERG P50 (Fig. 1d) or mfERG (Fig. 3b). Progressive degeneration encroaching upon the macula may lead to eventual blindness, and it is important to distinguish from other causes of rod system dysfunction. In RP, the DA 0.01 ERG is typically reduced and the bright flash (DA 3.0 and DA 10.0) ERGs show a-wave reduction. The reduction in the a-wave confirms rod photoreceptor dysfunction; there is concomitant b-wave reduction because the b-wave is generated “downstream” from the abnormal rod photoreceptors. The LA 30 Hz and LA 3.0 ERGs are typically delayed and/or reduced, but dysfunction is milder than in the rod system. The reduction in the a-wave makes the distinction from the two common forms of congenital stationary night blindness (complete and incomplete CSNB; see below). There are other rare forms of CSNB that cause severe rod-driven ERG abnormalities (DA 3.0 and DA 10.0 ERG a-wave reduction) but with spared cone system function, and these include “Riggs-type” CSNB, Oguchi disease and fundus albipunctatus. In the latter two disorders, there are usually characteristic fundus abnormalities and improvement or recovery of rod system function after prolonged DA (see Table 1 for a summary). The fundus appearance in fundus albipunctatus may be similar to patients with retinitis punctata albescens (Bothnia dystrophy); patients with Bothnia dystrophy may also show partial ERG recovery following prolonged dark adaptation, but the phenotype is more severe than in fundus albipunctatus and evolves to a progressive rod-cone dystrophy.

Acquired night blindness with a normal fundus can occur in vitamin A deficiency and CAR, although in rare cases of CAR there may be an electronegative ERG. The ERGs in vitamin A deficiency are characterized by severe rod system dysfunction and normal or near-normal cone system function, similar to the ERGs in “Riggs-type” CSNB. However, the ERGs in vitamin A deficiency usually return to normal following treatment.

#### Night blindness due to dysfunction occurring post-phototransduction

Complete and incomplete CSNB are associated with a normal fundus and generalized retinal dysfunction that is post-phototransduction (Fig. 1e, f), with normal (or near-normal) a-waves and electronegative DA 3.0 and DA 10.0 ERG waveforms (b/a ratio < 1). In complete CSNB, the DA 0.01 ERG is undetectable. The LA 30 Hz ERG, although often of normal amplitude, may have a slightly broadened trough and often shows borderline or mild peak time delay. The LA 3.0 ERG has normal a-wave amplitude but with a broadened bifid trough and a b-wave with a sharply rising peak with no oscillatory potentials; the b/a ratio varies but is usually mildly subnormal. The shape of the DA and LA ERG waveforms are characteristic of loss of On-pathway function with Off-pathway preservation, also evident in the long-duration On–Off ERG, which reveals an electronegative On response and a normal Off response. Complete CSNB is caused by a defect in 1 of 5 genes (Table 1), expressed by On-bipolar cells and consistent with the ERG abnormalities. In incomplete CSNB, the DA0.01 ERG is present but subnormal. LA 30 Hz ERGs are markedly reduced and have a bifid shape. The LA3.0 ERG is markedly subnormal with a low b:a ratio. Long-duration stimulation reveals reduction in both the On b-wave and Off d-wave. It is noted that the 2 genes implicated in incomplete CSNB (Table 1) are involved in neurotransmitter release from the photoreceptor presynaptic membrane, consistent with ERG evidence of both On- and Off-bipolar cell dysfunction.

Acquired night blindness with a normal fundus and electronegative ERG can occur in melanoma-associated retinopathy (MAR) and rarely in CAR (see above). MAR is rare but is associated with malignant melanoma, and the ERG findings are identical to those in complete CSNB (Fig. 1e). The ISCEV standard ERG features in MAR, CAR and vitamin A deficiency are different to each other, but are indistinguishable from some of the inherited disorders mentioned above, highlighting the importance of clinical context in the interpretation of ERGs.

### Photophobia

Photophobia is commonly associated with generalized cone system dysfunction and can be an early symptom in cone and cone-rod dystrophies. In cone dystrophies, the LA 30 Hz and LA 3.0 ERGs show delay and/or amplitude reduction, and in cone-rod dystrophy, there is additional abnormality of the DA ERGs (Fig. 1c). In both conditions, there is usually severe macular dysfunction evident on PERG (Fig. 1c) or mfERG testing. Rod monochromacy (achromatopsia) is characterized by severe cone system dysfunction from early infancy; the LA ERGs are typically undetectable, but the DA 0.01 ERG, selective for the rod system, is normal, and the DA 10.0 ERG is normal or shows mild reduction in the a- and b-waves, due to a loss of the normal dark-adapted cone system contribution. The ERG findings in S-cone monochromacy (a form of “X-linked incomplete achromatopsia”) are similar, but DA ERGs may be additionally attenuated due to high myopia; there may be a markedly abnormal (but detectable) LA 3.0 ERG and the short-wavelength (“blue”) flash ERG is relatively preserved. Congenital photophobia may also be a feature of albinism. Photophobia is rarely caused by dysfunction confined to the macula. Acquired causes of photophobia include retinal inflammatory disease such as uveitis and birdshot retinochoroidopathy (BRC), both associated with a high incidence of generalized cone system dysfunction, AIR and CAR. Photophobia is a rare feature of optic nerve disease but can also occur in neurological disorders such as migraine, meningitis and in carotid artery or vertebral artery disease.

### Visual field loss

Peripheral visual field constriction is a common feature of rod-cone dystrophy (RP), and this can occur without classical intraretinal pigment deposition, particularly in children. Cone and cone-rod dystrophies may present with visual field defects including central scotomata, generalized depression of sensitivity, ring scotomata and peripheral field loss if there is relative sparing of central macular function. Peripheral visual field loss may also occur in inflammatory retinal disorders such as BRC, associated with variable retinal dysfunction but often characterized by generalized cone system dysfunction, manifest as delay in the LA 30 Hz ERG, and sometimes associated with additional inner retinal rod system involvement (reduction in DA 10.0 ERG b:a ratio) which may be reversible following treatment (Fig. 1g, h). In acute zonal occult outer retinopathy (AZOOR), there is usually field loss disproportionate to visible fundus changes and persistent photopsia within the scotoma. Full-field ERG abnormalities are common, and some may show a reduction in the EOG light peak-to-dark trough ratio, not explained by abnormalities in rod function. Autoimmune disorders, such as CAR and AIR, may also present with rapid visual field constriction and marked ERG abnormality (see above). Homonymous hemianopic visual field defects usually reflect chiasmal or retrochiasmal brain lesions, and these may be detected by multichannel VEP recordings and require prompt further investigation. Field loss may also be seen in shallow retinal detachments and retinoschisis with concomitant full-field ERG changes, and clinical or ultrasound eye examination is essential.

### Disk pallor

Disk pallor may be a feature of optic neuropathy or retinopathy, including cone and cone-rod dystrophies. In central retinal artery occlusion (CRAO), there may be unilateral retinal edema and a “cherry red” spot at the fovea in the acute phase, but after a few weeks, this resolves as disk pallor develops. The subacute and chronic phases may be mistaken for ischemic optic neuropathy, and the electrophysiology enables the distinction. The ERG in CRAO has an electronegative DA 3.0 or DA 10.0 ERG, and there is usually marked involvement of the LA ERGs, in keeping with generalized inner retinal dysfunction. There are several other potential masquerades of optic neuropathy including occult maculopathy (inherited or acquired) and central serous chorioretinopathy (CSR); both may manifest PERG P50 or central mfERG abnormalities. In acute idiopathic blind spot syndrome (AIBSS), the mfERG may characterize the nasal area of reduced function (Fig. 3d). Occult retinopathy (including AZOOR), autoimmune and paraneoplastic retinopathies typically show marked ERG abnormalities, and in posterior scleritis, which like optic neuritis may be accompanied by pain on eye movement, there may be inflammatory changes affecting the retina that cause ERG abnormality.

### Glaucoma

Glaucoma is a progressive optic neuropathy associated with injury to retinal ganglion cell axons, frequently due to elevated intraocular pressure. Common signs include a characteristic pattern of optic atrophy (enlargement of the optic nerve cup), sectoral nerve fiber layer defects, often best visible with red-free light and evident on optical coherence tomography (OCT). There are often characteristic visual field defects, including arcuate “nerve fiber bundle defects” which reflect the distribution of optic nerve fibers emanating from the optic nerve, and “nasal steps” at the horizontal raphe.

The pattern ERG is sensitive to macular ganglion cell dysfunction and nerve fiber layer loss in glaucoma and can be of value in the evaluation of “glaucoma suspects” with glaucomatous risk factors such as elevated intraocular pressure, or optic nerve head changes, prior to the measureable loss of visual field. There may be reduction in the N95 (and also the P50) component in transient recordings, but steady-state PERG recordings are more affected. Traditional full-field ERG parameters, such as a-wave and b-wave amplitudes, are insensitive to ganglion cell injury, but there is increasing interest in the photopic negative response (PhNR). This is a late, cornea-negative deflection in the full-field ERG which is often recorded to red flashes presented on a blue background. The PhNR reflects global retinal ganglion cell function and offers the possibility of detecting and monitoring glaucomatous progression. ISCEV standard multifocal ERGs (first-order kernels) are driven primarily by photoreceptor and bipolar cells and are thus relatively insensitive to ganglion cell damage, although subtle effects of glaucoma have been described in the second-order kernels or with special stimulation paradigms. Multifocal recording technology has also been adapted to produce low-resolution visual field-like maps of VEP responses to spatial stimuli for eccentricities out to approximately 20° (e.g., dartboards), although standardization and clinical utility have yet to be established.

### Nystagmus

Congenital nystagmus is a feature of several ocular and neurological disorders. Isolated idiopathic congenital motor nystagmus (CMN) is not associated with other ocular or neurological abnormalities, and although pattern-reversal VEP and PERG may be difficult or impossible to record due to eye movements, flash VEPs and full-field ERGs are normal. Common retinal causes of nystagmus include Leber congenital amaurosis (LCA), associated with severe generalized photoreceptor dysfunction (DA and LA ERGs are severely reduced or undetectable), cone and cone-rod dystrophy, rod and S-cone monochromacy and complete and incomplete CSNB, characterized by different ERG phenotypes (see above). Nystagmus is also associated with ocular and oculo-cutaneous albinism (see above), and diagnosis in the former may be difficult in the absence of obvious skin depigmentation.

Acquired nystagmus may result from drug toxicity or medication that impairs the function of the labyrinth, thiamine or vitamin B12 deficiency, head injury, stroke, multiple sclerosis or any disease or injury of the brain that affects neural centers that control eye movements. Exclusion of afferent visual pathway dysfunction with electrophysiology may provide an important contribution to the management of such cases.

### Vascular retinopathies or ischemic status of retina

The full-field ERG is sensitive to retinal ischemic disorders affecting the inner retina. There may be reductions in the DA 3.0 and DA 10.0 ERG b:a ratios, the DA oscillatory potentials are usually abnormal or extinguished, and LA 30 Hz ERGs show prolonged peak times and waveform distortions. The ERG may be invaluable in detecting ischemic central retinal vein occlusion (CRVO), progression of non-ischemic to ischemic CRVO and in the diagnosis of ocular ischemic syndrome especially when the carotid Doppler scans are normal or equivocal. The ERG has advantages over commonly used fluorescein angiography in being safe and noninvasive, providing information on deeper layers and peripheral areas of retinal blood supply and may be informative in patients with systemic co-morbidities or pregnancy, in patients allergic to fluorescein dye or in cases of vitreous hemorrhage obscuring the fundus view. Prolonged LA 30 Hz ERG peak times are frequently seen in diabetic retinopathy and are associated with increased risk of disease progression. Peak time delays can be useful for screening, and loss of oscillatory potentials can occur in some diabetic patients without diabetic retinopathy and may identify patients at increased risk.

### Ocular media opacity

Full-field ERGs and flash VEPs can provide valuable information in patients with suspected retinal or visual pathway disease when the fundus is obscured or when the use of retinal imaging techniques is precluded by an opaque ocular media. Integrity of retinal and visual pathway may be important considerations prior to treating patients with corneal lesions, cataracts or vitreous hemorrhage, particularly if there is a history of retinal detachment, retinal or neurological involvement. A normal or relatively preserved ERG or flash VEP may suggest a better prognosis for improved vision. An abnormal full-field ERG may suggest generalized retinal dysfunction but may also occur in vitreous hemorrhage. An abnormal ERG does not exclude central visual recovery because it does not assess macular function. It is noted that the ERG is usually abnormal in the presence of intraocular silicone oil tamponade (for retinal detachment), but interpretation is confounded because the oil impedes conduction of the electrical signals from the retina to the corneal surface.

### Family history of visual pathway disease

Visually asymptomatic patients with a family history of retinal or optic nerve disease or suspected cases of syndromic retinal dystrophy may require electrophysiological testing for evidence of subclinical disease. For example, visually asymptomatic obligate carriers of X-linked RP usually manifest abnormal and asymmetrical ERG abnormalities, irrespective of whether there is fundus abnormality, whereas the ERGs in carriers of X-linked choroideremia are usually normal until late in life. Carriers of X-linked ocular albinism and patients with rubella retinopathy may also have abnormal fundus pigmentation; the ERGs are normal in the former and normal or near-normal in the latter. There is variable expressivity in (autosomal dominant) Best disease such that some heterozygotes have a normal fundus and an EOG may be needed to confirm the diagnosis. Similarly, VEP and PERG N95 abnormalities may indicate optic nerve and retinal ganglion cell dysfunction in cases of suspected dominant optic atrophy.

### Monitoring of disease progression, treatment efficacy and safety

Serial testing may assist the distinction between stationary and progressive conditions, important for diagnosis and patient counseling. Pattern and flash VEPs have diverse applications and may be used to monitor visual pathway maturation in infants with poor vision or amblyopia or to monitor optic nerve function in patients with known neurological disease. In inflammatory retinal diseases such as BRC, the ERGs can be used to monitor efficacy of treatment objectively (Fig. 1g, h), thus informing clinical management and titration of potentially toxic medication. Worsening VEPs may prompt the need for surgical intervention in dysthyroid eye disease or in neurological disorders, irrespective of stable neuroradiology. Several medications commonly administered systemically for non-ocular conditions are potentially toxic to the macula, retina or optic nerves, and pre-treatment assessment and monitoring may be considered. The multifocal ERG, for example, may reveal annular or parafoveal macular dysfunction that can manifest as an early stage of hydroxychloroquine toxicity, before the development of a visible “bulls-eye” lesions and before structural changes are evident or obvious on retinal imaging. Intraocular drugs, intraoperative dyes and bright lights of ophthalmic surgical equipment have become another source of toxic/phototoxic maculopathy that may need retinal and macular electrophysiology testing for monitoring, for clinical evaluation or for diagnosis. ERG evaluations are also becoming an integral part of various clinical trials comparing outcome efficacies of various surgical or medical procedures involving the macula such as macular holes, epiretinal membranes, anti-VEGF treatments, macular detachments and central serous chorioretinopathy. Similarly, ERGs may be used to monitor retinal safety of new treatments and as objective outcome measures in clinical trials that aim to restore visual function or arrest disease progression.

## Special considerations and indications for ERG and VEP testing in infants and children

Accurate diagnosis may be difficult in young children who are unable to describe their visual symptoms or who are difficult to examine. The objective data provided by electrophysiological testing are fundamental to the management of the child with suspected visual pathway dysfunction, but there are important considerations relating to maturation of responses, ability to comply with testing and causes of visual pathway dysfunction more specific to the pediatric population. Both ERG and VEP responses show profound developmental changes during infancy and childhood, and although all visual electrophysiological values are considered in relation to age, it is even more important in young patients. Infants up to the age of about 2 years can frequently undergo successful ERG testing without general anesthesia, while being held in a parent’s lap, either by using only topical anesthetic eye drops and corneal electrodes or by using surface electrodes on the lower eyelids. It may be appropriate to shorten the standard ERG protocol, and many practitioners start with light-adapted ERGs and perform limited dark adaptation, dependent upon the compliance and comfort of the child. VEP testing in infants is equally feasible, but may require simple flash stimulation, if steady fixation on the center of the VEP pattern stimulus cannot be induced with a moving toy, jangling keys or similar to encourage central fixation.

Examination under anesthesia may enable the use of corneal electrodes in the non-compliant child, but anesthesia usually alters ERG timing and amplitudes, and interpretation requires caution. Similarly, the use of skin electrodes limits sensitivity since the signal amplitude is lower, but in this age group there is rarely a need to detect subtle abnormalities and most clinically appropriate questions may be easily addressed. For example, is there a detectable ERG, is there a functioning cone system, is there a response after dark adaptation and is there an electronegative ERG waveform? The cortical neurons which drive the VEP are much more susceptible to general anesthesia than the retina, precluding reliable VEP recordings.

### Unexplained visual loss

Absent or impaired visually mediated behavior may indicate a disorder affecting any level of the visual system. Babies who do not fix and follow and presumed amblyopic patients that fail to respond to treatment may require testing to confirm or exclude pathology. Early diagnosis of retinal dystrophy may be essential to identify young candidates who are potentially amenable to future experimental treatments. A normal ERG may also prompt the need for further investigations such as VEPs or neuroradiology. Non-organic visual loss is relatively common in older children, and in such circumstances, the electrophysiological data are usually normal even though there may be reported profound visual loss.

### Congenital nystagmus

The differential diagnosis includes several retinal disorders such as Leber congenital amaurosis, congenital stationary night blindness, and rod and S-cone monochromacy. The ERG will help differentiate these conditions. Young children with albinism show multichannel flash VEP evidence visual pathway misrouting, although with increasing age (above about 5 years) this may be best demonstrated with pattern onset–offset VEPs. Flash VEPs and ERGs are normal in idiopathic CMN. Clinical examination is also needed to investigate or exclude TORCH infections like viral retinitis that result in nystagmus and variable ERG abnormalities.

### Known or suspected hereditary disorders

The ERG may be helpful in advising families with patients at risk of hereditary retinal disorders. The extent to which the various retinal dystrophies are detectable in early infancy is frequently not known, but a normal ERG at age 7 or 8 years of age largely excludes X-linked RP. Night blindness may be associated with RP or CSNB and ERGs help differentiate between progressive and stationary disorders. In young cases of suspected Best disease, children may be unable to comply with EOG testing, but testing of the parents will almost invariably identify the parent carrying the mutation, irrespective of whether the fundi are normal.

### Perinatal infections

Perinatal infections, particularly the “TORCH” agents, may attack ocular tissues, with possible profound associated dysfunction. The most common perinatal infection is probably rubella retinopathy, which frequently results in mottled RPE pigmentation ad a “salt and pepper” appearance, but in such cases the ERG is usually normal or near-normal.

### Perinatal brain injury

Perinatal brain damage may lead to severe visual impairment. VEPs enable objective determination of the nature of the deficit and may help grade the severity of cortical dysfunction. However, it is important to recognize that VEPs do not reflect higher processing required for normal vision.

### Trauma

In children who have suffered head/orbital trauma or suspected visual pathway injury, complementary retinal and VEP testing may localize dysfunction and help to confirm, exclude or distinguish between retinopathy and optic nerve or post-retinal dysfunction, particularly in those unable or too young to communicate verbally.

### Delayed visual maturation

Infants often present with “visual indifference,” showing little or no reaction to visual stimuli for several months. If the eye examination, ERG, and VEP are normal or near-normal, this provides reassurance, and the prognosis for development of normal or near-normal vision is reasonably good.

### Monitoring for retinal drug toxicity

The most common indication in this category is vigabatrin, which is used for the treatment of infantile spasms (West syndrome). The drug causes peripheral visual field constriction in approximately 30% of adults. The ERG is helpful in monitoring patients who are too young or lack the ability to perform visual field testing.

### Amblyopia

Children suspected of having amblyopia are often referred for electrophysiology to exclude other causes of poor vision, for example when visual acuity has not improved with patching and the fundi are normal or when visual acuity is reduced bilaterally. In amblyopic eyes, pattern-reversal VEPs may show amplitude reduction; delays in the major positive (P100) component can occur, but this tends to be more prominent in strabismic rather than anisometropic amblyopia. Pattern VEPs may also be used to monitor the efficacy of occlusion therapy in amblyopic and fellow eyes, but subjective assessment of vision (if possible) should generally take priority.

## Complementary testing

Electrophysiological testing complements routine ophthalmic examination, subjective tests of visual function and retinal imaging methods commonly employed in the assessment of patients with visual impairment. Electrophysiological methods are objective and uniquely assess aspects of function and dysfunction. Ophthalmic examination and imaging techniques may be normal in the presence of retinal and visual pathway dysfunction or may reveal abnormalities that do not correlate with the nature or severity of dysfunction. Optimal assessment is obtained with judicious use of widely used techniques including those outlined below.

### Subjective assessment of function

#### Visual acuity

Visual acuity (VA) testing is a long established method of assessing central visual function in almost any form of visual system pathology, from ptosis of the eyelids and corneal epithelial edema to retinal degenerations and optic neuropathies. However, VA loss is non-specific and cannot be used to localize dysfunction within the visual pathway. The VA does not give an indication of peripheral retinal function and may also be relatively or completely preserved in the presence of macular or optic nerve dysfunction. VA may be normal, for example, in paracentral and peripheral retinal derangements, nerve fiber bundle defects (as in glaucoma), in subacute optic neuritis and lesions of the posterior visual pathways which spare the projections of the central retina.

#### Visual fields

Visual field testing is widely available and, with the advent of automated static perimetry, highly standardized and reproducible. Visual fields allow localization of visual impairment, with classic patterns of visual field loss associated with localized and generalized retinal disorders, macular and optic nerve disease, chiasmal disruptions, lesions of the lateral geniculate body and optic radiations, and cortical lesions. Pattern ERG and multifocal ERG can be of great value in distinguishing between macular and optic nerve disease, often associated with similar visual field abnormalities and often indistinguishable by VEP alone. Full-field ERG abnormalities are a leading indicator of degenerative retinal disorders such as retinitis pigmentosa. Peripheral visual fields are important in the adequate assessment of degenerative retinal diseases such as retinitis pigmentosa, in which the extent of scotomas and the presence of residual temporal islands of vision are of great importance to the patient but cannot be adequately assessed by central Humphrey visual fields and peripheral automated visual field protocols. It is highlighted that visual fields do not always correlate with objective suprathreshold electrophysiological measures of function.

#### Contrast sensitivity

Loss of contrast sensitivity is readily documented with special eye charts designed for the task, or CRT-based vision testing devices, and can occur in the absence of significant VA reduction. The causes of reduced contrast sensitivity include optical problems such as corneal haze or cataract, and complementary use of different electrophysiological tests (Fig. 4) can differentiate these from a wide range of visual pathway disorders.

#### Color vision testing

Color vision is an important visual faculty, and abnormalities may derive from retinal, optic nerve or (rarely) cortical pathology. Commonly used Ishihara plates are highly sensitive to even minor dyschromatopsias, but detect only red-green (protan or deutan axis) abnormalities. Other sets of test plates, such as the H-R-R plates, also detect tritan axis problems. The common X-linked protan and deutan color vision defects are rarely associated with abnormalities in the ISCEV standard ERG, but can be detected with nonstandard chromatic stimuli. Absence or severe loss of normal color vision suggests more severe pathology, such as achromatopsia or optic nerve disease, which are readily detected by ERG or VEP.

#### Dark adaptometry

Abnormalities of dark adaptation are difficult for patients and physicians to assess without formal testing, as normal difficulties seeing in dim light may be reported as abnormally impaired night vision. Formal dark adaptometry can be performed with specialized instruments, such as the Goldmann–Weekers Dark Adaptometer. Qualitative assessment can be readily obtained with much simpler materials, such as the Hyvarinen cone adaptation test, in which an examiner with normal dark adaptation compares his/her adaptation with that of the patient, who is asked to sort colored plastic tiles in a very dim room. Abnormalities of dark adaptation generally imply retinal pathology, including CSNB, vitamin A deficiency, paraneoplastic retinopathies and degenerative disorders such as retinitis pigmentosa, usually readily differentiated by full-field ERG in clinical context.

### Retinal imaging

Fundus photography has been available as a clinical tool since 1926, and fluorescein angiography was introduced in 1959. More recently, advances in fundus imaging have appeared with increasing frequency, not only documenting ophthalmoscopic findings, but extending the range of clinical perception in depth (ICG angiography) and resolution; spectral domain OCT now approaches the resolution of low-power microscopy, without the need to remove tissue from the eye for histologic processing. However, the enhanced capability of fundus imaging has not displaced electrophysiological methods of testing function. The need to complement anatomical methods with studies of visual function is as keen as ever and perhaps more so as increasing detail in fundus imaging allows ever finer diagnostic distinctions to be made, for which the functional consequences must be determined.

#### Fundus photography

Fundus photography documents the appearance of the retina and allows rapid estimation of the size and characteristics of fundus lesions. Digital photography has improved resolution and enabled more objective assessments as well as multi-spectral imaging. Newer cameras provide a wider-field image far greater than the 30°–40° fields of traditional fundus cameras, revealing important pathology of the peripheral retina which was previously unappreciated or more difficult to assess, especially in children.

#### Fluorescein angiography

Fluorescein angiography documents the extent and integrity of the retinal vasculature and remains an important tool even in the era of advanced OCT imaging, which lacks the dynamic aspect of the evolving fluorescein angiogram. ICG angiography extends the range of angiographic imaging deeper into the choroid, demonstrating vascular structures and abnormalities that may be less evident or undetectable using other methods.

#### Fundus autofluorescence

Fundus autofluorescence imaging (FAF) can reveal otherwise invisible manifestations of disrupted RPE metabolism. The main fluorophore to short-wavelength excitation is lipofuscin, derived from the phagocytosis of shed photoreceptor outer segments in the RPE. The distribution of FAF across the posterior pole and abnormal accumulations or depletions of the FAF signal can detect or accentuate the appearance of lesions in a wide range of disorders, and the technique has largely replaced fluorescein angiography in the assessment of inherited retinal and macular dystrophies. Since the technique was introduced in the early 1990s, methods such as PERG and mfERG have helped establish the functional significance of common FAF abnormalities and the value of FAF in monitoring disease progression.

#### Optical coherence tomography

Optical coherence tomography (OCT) has revolutionized retinal evaluation. It is far superior to even the most careful ophthalmoscopy at detecting anatomical disruptions of the posterior pole, such as cystoid edema, vitreomacular traction or shallow serous detachments of the retina or RPE. Moreover, the recognition of the role of the line of photoreceptor inner segment ellipsoid (or inner segment/outer segment junction) as an indicator of the integrity of the photoreceptors has clarified the diagnosis of many retinal disorders. For example, in many cases of “occult macular dystrophy” OCT may expose subtle or localized outer retinal loss. Focal OCT abnormalities do not always correlate with the severity of dysfunction or the function of surrounding retinal tissues.

#### Adaptive optics

Adaptive optics (AO) techniques use active optical elements to compensate for the optical aberrations of the eye and provide a noninvasive method for extending spatial resolution and studying the micro-morphology of the retina in vivo. Clinical implications are only beginning to emerge, but otherwise invisible disruptions in the photoreceptor mosaic have been documented in different retinal and macular disorders.

### Genetic testing

Electrophysiology has a pivotal role to characterize disorders and the phenotypic variability associated with a known genotype or to guide the screening of genes associated with a known electrophysiological phenotype. Advances in molecular biology have enabled genotyping of many inherited retinal and macular dystrophies, but the functional consequences remain difficult to predict due to allelic heterogeneity, genetic modifiers and other factors. In rare retinal dystrophies, ERGs can be used to identify the gene responsible, e.g., in enhanced S-cone syndrome (NR2E3), “cone dystrophy with supernormal ERG” (KCNV2) and RGS9/R9AP-retinopathy, as outlined in Table 1. It is more usual for the ERGs to suggest a range of disorders or possible genotypes, e.g., in complete CSNB, the ERG phenotype is common to X-linked and autosomal recessive forms with mutations in 1 of several different genes and ERGs are additionally identical to those in melanoma-associated retinopathy, highlighting the importance of interpretation in clinical context. The emergence of unbiased whole exome and whole genome sequencing may reveal novel or unexpected genetic alterations and electrophysiological interrogation likely to prove increasingly important to establish the functional consequences and genotype–phenotype correlations.

**Table 1:** Typical electrophysiological abnormalities in selected retinal and visual pathway disorders

	Typical or common fundus/ocular abnormalities	Acquired disorder or gene/s implicated^a^	Macular function	Rod system function		Cone system function		Comments including VEP, EOG and other electrophysiological findings where relevant
			PERG P50 or MfERG	DA 0.01	DA10.0	LA 30 Hz	LA 3.0	
Adult vitelliform macular dystrophy	Small vitelliform foveal lesion due to a sub-retinal cyst with or without paracentral drusen and mild RPE changes	PRPH2, BEST1, IMPG1, IMPG2	N/A	N	N	N	N	The EOG is normal or mildly subnormal and distinguishes most cases from Best vitelliform macular dystrophy
Albinism	Blonde fundus and foveal hypoplasia are common. There may be iris transillumination and nystagmus	TYR, OCA2, TYRP1, SLC45A2, GPR143	A	N	N	N	N	Multichannel VEPs show bilateral contralateral predominance to pattern onset–offset (adults) or flash stimulation (young children). Assessment of macular function may be precluded by the effects of nystagmus; in the absence of nystagmus there may be evidence of mild macular dysfunction in some cases
Autosomal dominant vitreoretinochoroidopathy (ADVIRC)	Liquefied vitreous. Preretinal white dots and neovascularization often present. Peri-papillary atrophy can occur. Abnormal pigment often extends to an equatorial demarcation line at the posterior border	BEST1	A	A	A	A	A	The EOG light peak-to-dark trough ratio is severely abnormal
Autosomal recessive bestrophinopathy (ARB)	Diffuse RPE irregularity extending to the vascular arcades associated with patchy RPE atrophy and punctate white dots	BEST1	A/N	A/N	A/N	A/N	A/N	The EOG light peak-to-dark trough ratio is severely abnormal. ERG is initially normal but mild abnormalities usually develop in late childhood or adolescence and then worsen progressively
Batten disease (Juvenile onset neuronal ceroid lipofuscinosis)	Normal or Bull’s eye lesion	CLN3	A	A	A (-ve)	A	A (-ve)	Electronegative ERG may be detected before fundus changes. LA 3.0 ERG may have a low b:a ratio
Birdshot retinochoroidopathy (BRC)	Multiple pale sub-retinal lesions. Inflammatory signs such as vitritis, vasculitis and CME are common	Acquired	A/N	A/N	A(-ve)/N	A/N	A/N	Variable. Mf ERG and PERG often reveal macular dysfunction, especially if there is CME. ERG is normal to abnormal depending on severity and efficacy of treatment. LA 30 Hz ERG commonly delayed and DA strong flash ERGs in some cases have a low b:a ratio
Best vitelliform macular dystrophy (Best disease)	Variable but often characterized by a vitelliform yellow macular lesion due to a sub-retinal cyst, which may evolve in some to become vitelliruptive with eventual atrophy. Fundus can be normal	BEST1	N/A	N	N	N	N	The EOG light peak-to-dark trough ratio is abnormal. Macular dysfunction occurs as macular lesions become vitelliruptive
Bulls-eye maculopathy (BEM)	Concentric paracentral changes with foveal sparing	Acquired or Genetic	A A A	N N A	N N A	N A A+	N A A+	Maculopathy or macular dystrophy Cone dystrophy Cone-rod dystrophy PERG/mfERG evidence of macular dysfunction; mfERG may reveal paracentral dysfunction with localized or relative foveal sparing
Carcinoma Associated Retinopathy (CAR)	Fundus initially normal. RPE atrophy, mottling and vessel attenuation may develop	Acquired	A	A+	A+	A+	A+	Often severe photoreceptor dysfunction causing an undetectable ERG or severe a-wave reduction. Cone system is most affected in some. In rare cases there is an electronegative ERG
Central retinal artery occlusion (CRAO)	Inner retinal edema and a cherry red spot at the macula in the early stages. Eventual arteriolar attenuation and disk pallor	Acquired	A (variable)	A	A (-ve)	A	A	Decreased oscillatory potentials. Relative sparing of visual acuity and of the PERG/central mfERGs if there is a cilioretinal artery
Central retinal vein occlusion (CRVO)	Dilatation and tortuosity of retinal veins, dot and flame hemorrhages, cotton wool spots, optic disk and macular edema, hyperemia. Ischemic form may result in severe vascular leakage and rubeosis	Acquired	A (variable)	A	A(-ve)	A	A	Reduced oscillatory potentials. Ischemic CRVO associated with more severe ERG changes and a more reduced DA ERG b:a ratio than non-ischemic disease. ERG a-wave involvement in severe cases
Choroideremia	Loss of RPE and choriocapillaris. Inner retina and optic disk normal. Late involvement of macula.	REP1	A	A+	A+	A	A	Severe (+) rod > cone or undetectable ERGs. Late macular involvement. ERG is usually normal in female heterozygotes but worsening can occur from middle age
	Female heterozygotes may show mild pigmentary changes or patchy RPE degeneration		N	N	N	N	N	
Cone dystrophy	Fundus may be normal. Disk pallor, granular RPE, bull’s eye lesion, central atrophy	see Ret Net (many)	A	N	N	A	A	See text. PERG and MfERG usually show evidence of severe and early macular involvement
Cone-rod dystrophy	Fundus may be normal. Disk pallor, granular RPE, bull’s eye lesion, central atrophy.		A	A	A	A+	A+	
CSNB 1. Schubert–Bornschein								
(a) complete	Fundus normal (± myopic changes).	NYX, GRM6, TRPM1, LRIT3, GPR179	A	U	A (−ve)	A	A	See text for details
(b) incomplete	Fundus normal (± myopic changes).	CACNA1F, CABP4	A	A	A (−ve)	A+	A+	
2. Riggs-type	Fundus normal	PDE6B, RHO, GNAT1, SLC24A1	N	A	A	N	N	See also fundus albipunctatus and Oguchi disease (forms of CSNB with abnormal fundi and delayed dark adaptation)
Dominant optic atrophy (DOA)	Disk pallor typically wedge shaped and temporal but may be diffuse	OPA1, OPA3, OPA4, OPA5, OPA8	PERG P50 N or mildly subnormal and of short peak time MfERG N	N	N	N	N	PERG N95 may be abnormal in early stages. In severe cases PERG P50 may be reduced with shortening of P50 peak time. Pattern VEP often shows delay and reduction but abnormalities can be mild in the early stages
Enhanced S-cone syndrome (Goldman Favre disease)	Normal to nummular pigment clumping in RPE in vicinity of vascular arcades. Macular schisis can occur	NR2E3	A	U	A	A+	A	Pathognomonic ERG abnormalities. DA3, DA10 and LA3 ERGs are severely delayed with a simplified waveform. LA3 ERG a-wave is larger than the severely abnormal LA 30 Hz ERG. S-cone ERG is enlarged
Fundus albipunctatus	Multiple small white/yellow spots with sparing of the macula	RDH5	A/N	A	A	A/N	A/N	See text. DA ERGs improve or normalize after prolonged dark adaptation. Approximately 50% have mild LA ERG abnormalities
Glaucoma	Disk cupping, nerve fiber loss	Acquired	PERG abnormal MfERG normal	N/A	N/A	N/A	N/A	VEP may be normal or mildly abnormal unless severe/advanced disease. Steady-state PERG more sensitive than transient PERG for monitoring purposes. PhNR may be used to assess global retinal ganglion cell function
Ischemic optic neuropathy.	In arteritic form optic disk swelling, disk pallor ± flame hemorrhages	Acquired	PERG P50 N or mildly subnormal and of short peak time MfERG N	N	N	N	N	Pattern VEPs show reduction without significant delay. More severe in arteritic (AAION) than non-arteritic (NAION) cases. There may be eventual PERG N95 reduction in keeping with retinal ganglion cell dysfunction and with reduction/shortening of P50 peak time in some. Usually unilateral
KCNV2-retinopathy (“Cone dystrophy with supernormal rod ERG”)	Normal in young but BEM and macular RPE atrophy may develop. Disk pallor in some. Peripheral retina normal	KCNV2	U	A	A	A	A	Pathognomonic ERG abnormalities. Generalized cone system dysfunction with unusual rod system involvement; DA ERGs to dim flashes are small and delayed and ERG b-waves to strong flashes large. DA 10 ERG a-wave has a distinctive broad trough with a late negative component
Leber congenital amaurosis (LCA)	Pigmentary & atrophic changes with age. Hypoplastic/swollen disks common	see Ret Net (many)	A	A+	A+	A+	A+	ERG typically undetectable or severely reduced from early infancy
Leber hereditary optic neuropathy (LHON)	Nerve fiber layer swelling in acute stages. Enlarged or telangiectatic and tortuous peri-papillary vessels. Optic atrophy	G11778A, T14484C, G3460A	PERG P50 N or mildly subnormal and of short peak time MfERG usually N	N	N	N	N	PERG N95 may be abnormal in acute stage. Pattern VEPs are undetectable or severely abnormal. Absence of fluorescein leakage from the swollen disk, distinguishing LHON from other forms of disk swelling.
Melanoma-associated retinopathy (MAR)	Fundus usually normal. Vitreous cells, vessel attenuation and disk pallor may develop in some	Acquired	A	U	A(-ve)	A	A	Long-duration On–Off ERG shows On response b-wave reduction with sparing of the Off -response. Full-field ERGs identical to those in complete CSNB
Oguchi disease	Golden fundus sheen which resolves following prolonged dark adaptation (Mizuo–Nakamura phenomenon)	SAG, rhodopsin kinase	N	U	A	N	N	DA ERGs show severe rod dysfunction after 20 min in the dark. LA ERGs are normal. After prolonged DA a single strong flash elicits a normal ERG; subsequent flashes elicit subnormal responses and further prolonged DA is needed to recover
Optic neuritis	Disk pallor and thinning of retinal nerve fiber layer may be evident	Acquired	PERG P50 N or mildly subnormal and of short peak time MfERG N	N	N	N	N	Pattern VEP is usually delayed with or without amplitude reduction. PERG P50 is usually normal but in 35% cases there is PERG N95 reduction in keeping with retinal ganglion cell dysfunction and with reduction/shortening of P50 peak time in some. May be subclinical involvement of the other eye
Pattern dystrophy	Various patterns of pigment deposition within the macula including adult-onset vitelliform macular dystrophy, butterfly-shaped, reticular, multifocal pattern dystrophies and fundus pulverentulus	PRPH2	A/N	N	N	N	N	The EOG is normal or mildly subnormal. The ERG is usually normal although there can be marked variability in fundus appearance and ERG phenotype within families with PRPH2 mutation
Retinitis Pigmentosa (RP; Rod-cone dystrophy)	Classically bone-spicule formation, RPE atrophy, attenuated vessels, disk pallor. Normal or near-normal in some	see Ret Net (many).	A/N	A+	A+	A	A	See text. Rod-cone dystrophy of variable severity. Variable macular involvement. In X-linked pedigrees, female heterozygotes usually have ERG abnormalities with inter-ocular ERG asymmetry
Rod monochromacy (“Achromatopsia”)	Usually normal, macular granularity may develop	CNGA3, CNGB3, GNAT2, PDE6C, PDE6H, ATF6	A	N	N/sl. A	U	U	See text. There may be mild reduction in DA strong flash ERGs due to loss of the normal cone system contribution to the a- and b-waves
Retinal toxicity (selected examples)	Chloroquine/Hydroxychloroquine	Acquired	A	N/A	N/A	N/A	N/A	MfERG shows annular macular dysfunction in early stages with later central involvement. ERG abnormal in severe cases
	Desferrioxamine		A	N/A	N/A	N/A	N/A	Macular dysfunction most common; PERG/mfERG ± ERG abnormality. ERG may be normal but ranges from showing mild rod dysfunction to severe cone-rod dysfunction
	Quinine		A	A	A(-ve)	A	A	On response electronegative; Off d-wave has an abnormal shape
Retinitis punctata albescens (Bothnia dystrophy; rod-cone dystrophy)	Multiple small white/yellow spots with sparing of the macula. Diffuse RPE degeneration, scalloped peripheral atrophy and pigment deposition in late stages	RLBP1	A/N	A	A	A	A	Resembles fundus albipunctatus in early stages and DA ERGs may show partial improvement after prolonged dark adaptation. Eventual progressive rod-cone dystrophy
RGS9 / R9AP – retinopathy (“Bradyopsia”)	Fundus normal	RGS9, R9AP	PERG U	N	SI. A	U	A+	DA 10 ERG mildly abnormal unless inter-stimulus interval is increased e.g. to 1-2 mins. Scotopic red flash ERG reveals normal rod and good DA cone function, in spite of severe LA ERG abnormalities.
S-cone monochromacy (“X-linked incomplete achromatopsia”)	Usually normal, macular granularity may develop	OPN1LW, OPN1MW	A	N	N/sl. A	U	A	A preserved S-cone ERG distinguishes the disorder from rod monochromacy. There may be relatively mild reduction in DA 0.01 and DA10 ERG a-waves due to high myopia and loss of cone system contribution to the strong flash ERG a- and b-waves
Stargardt disease/fundus flavimaculatus (ABCA4-retinopathy)	Central atrophy with flecks or widespread flecks across posterior pole with peri-papillary sparing. Extensive RPE atrophy in severe cases	ABCA4	A A A	N N A	N N A	N A A	N A A	Macular dystrophy Cone dystrophy Cone-rod dystrophy In all 3 phenotypes there is PERG/mfERG evidence of macular dysfunction
Vitamin A deficiency	Normal or white spots across the fundus	Acquired	N	A	A	N	N	See text.
X-linked retinoschisis	Macular cysts common; may progress to macular atrophy in older men. Peripheral schisis occurs in about 50% of cases	RS1	A	A	A(-ve)	A	A	On b-wave ± OFF d-wave subnormal. Inner retinal dysfunction of variable severity. PERG and mfERG usually abnormal

“-ve” signifies an electronegative ERG; b-wave that is smaller than a normal or near-normal a-wave (b:a < 1)

*N* normal, *A* abnormal, *sl. A* slightly abnormal, *A*+ severely abnormal, *U* undetectable

^a^See RetNet, the Retinal Information Network for details of disease genes and updates on disease genes; RetNet, http://www.sph.uth.tmc.edu/RetNet/

## Index

**Table:** 

**A**	
Achromatopsia (rod monochromacy)	12, 17, 23, 24
Acute zonal occult outer retinopathy (AZOOR)	12, 13
Adult vitelliform macular dystrophy	10, 19
Albinism	12, 13, 14, 15, 19
Amblyopia	14, 16
Arteritic anterior ischemic optic neuropathy (AAION)	10, 22
Autoimmune retinopathy (AIR)	10, 12, 13
Autosomal dominant vitreoretinochoroidopathy (ADVIRC)	9, 19
Autosomal recessive bestrophinopathy (ARB)	9, 19
**B**	
Batten disease (juvenile onset neuronal ceroid lipofuscinosis)	19
Best vitelliform macular dystrophy (Best disease)	10, 20
Birdshot retinochoroidopathy (BRC)	2, 12, 14, 19
Bulls-eye maculopathy	9, 14, 19, 20
**C**	
Carcinoma Associated Retinopathy (CAR)	10, 11, 12, 20
Central retinal artery occlusion (CRAO)	12, 20
Central retinal vein occlusion (CRVO)	13, 20
Central serous chorioretinopathy (CSR)	12, 14
Chiasmal dysfunction	8, 10, 17
Chloroquine	23
Choroideremia	14, 20
Compressive lesions	10
Cone & cone-rod dystrophy	2, 9, 12, 13, 18, 20, 22
Congenital nystagmus	13, 15
Congenital Stationary Night Blindness (CSNB)	2, 11, 13, 15, 17, 18, 21
**D**	
Delayed visual maturation (DVM)	16
Demyelination	10
Desferrioxamine	23
Disc pallor	12, 20, 21, 22, 23
Dominant optic atrophy (DOA)	10, 14, 21
**E**	
Enhanced S-cone syndrome	18, 21
Ethambutol	10
**F**	
Fundus albipunctatus	11, 21, 24
Fundus flavimaculatus (ABCA4-retinopathy)	9, 24
**G**	
Glaucoma	13, 16, 21
**H**	
Hydroxychloroquine	9, 14, 23
**I**	
Ischemic optic neuropathy (AAION; NAION)	6, 10, 12, 22
**J**	
Juvenile onset neuronal ceroid lipofuscinosis (Batten disease)	19
**K**	
KCNV2-retinopathy (Cone dystrophy with supernormal rod ERG)	18, 22
**L**	
Leber congenital amaurosis (LCA)	13, 15, 22
Leber hereditary optic neuropathy (LHON)	10, 22
**M**	
Macular dystrophy/maculopathy	2, 6, 7, 9, 10, 12, 14, 18, 19, 20, 24
Melanoma Associated Retinopathy (MAR)	11, 12, 18, 22
Methyl-alcohol poisoning	10
**N**	
Night blindness	11, 15, 17
Non-arteritic anterior ischemic optic neuropathy (NAION)	6, 10, 22
Non-organic visual loss	10
Nystagmus	8, 13, 15, 19
**O**	
Occult macular dystrophy/occult maculopathy	9, 10, 12, 18
Oguchi disease	11, 22
Optic nerve dysfunction	6, 8, 9, 10, 13, 14, 16
Optic neuritis	6, 8, 10, 13, 16, 23
**P**	
Paraneoplastic retinopathy (CAR, MAR)	10, 11, 12, 13, 17, 20, 22
Pattern dystrophy	10, 23
Perinatal brain injury	16
Perinatal infection	15
Photophobia	12
Photopic negative response (PhNR)	13, 21
Phototoxic maculopathy	14
**Q**	
Quinine	23
**R**	
Retinal and RPE disorders	9
Retinal detachment	12, 14, 18
Retinal toxicity	14, 16, 23
Retinitis Pigmentosa (RP; rod cone dystrophy)	2, 6, 11, 12, 14, 15, 17, 23
Retinitis punctata albescens (Bothnia dystrophy)	11, 24
Retrochiasmal dysfunction	8, 10, 12
RGS9/R9AP-retinopathy	18, 24
Rod monochromacy (achromatopsia)	12, 13, 15, 23
Rubella retinopathy	14, 16
**S**	
S-cone monochromacy (X-linked incomplete achromatopsia)	12, 13, 15, 24
Silicone oil	14
Stargardt disease (ABCA4-retinopathy)	9, 24
**T**	
Tobacco toxicity	10
TORCH	15
Trauma	16
**U**	
Unexplained visual loss	10, 15
**V**	
Vascular Retinopathies	12, 13, 20
Vigabatrin	16
Vitamin A deficiency	11, 12, 17, 24
Vitamin B12 deficiency	10, 13
**X**	
X-linked retinoschisis	12, 24
X-linked RP	14, 15
